# Microglial Mayhem NLRP3 Inflammasome's Role in Multiple Sclerosis Pathology

**DOI:** 10.1111/cns.70135

**Published:** 2024-12-17

**Authors:** Hua Fan, Qizhi Fu, Ganqin Du, Ling Qin, Xiaofei Shi, Dongmei Wang, Yanhui Yang

**Affiliations:** ^1^ Office of Research & Innovation The First Affiliated Hospital, and College of Clinical Medicine of Henan University of Science and Technology Luoyang China; ^2^ Department of Intensive Medicine The First Affiliated Hospital, and College of Clinical Medicine of Henan University of Science and Technology Luoyang China; ^3^ Department of Neurology The First Affiliated Hospital, and College of Clinical Medicine of Henan University of Science and Technology Luoyang China; ^4^ Department of Hematology The First Affiliated Hospital, and College of Clinical Medicine of Henan University of Science and Technology Luoyang China; ^5^ Department of Rheumatology and Immunology The First Affiliated Hospital, and College of Clinical Medicine of Henan University of Science and Technology Luoyang China; ^6^ School of Basic Medical Sciences Henan University of Science and Technology Luoyang China; ^7^ Department of Emergency Medicine The First Affiliated Hospital, and College of Clinical Medicine of Henan University of Science and Technology Luoyang China

**Keywords:** experimental autoimmune encephalomyelitis, inflammasome, macrophages, microglia, multiple sclerosis, NLRP3

## Abstract

**Introduction:**

This review delves into the intricate relationship between NLR inflammasomes, particularly the NLRP3 inflammasome, and the immune‐mediated neurodegenerative disease, multiple sclerosis (MS). While the precise etiology of MS remains elusive, compelling research underscores the pivotal role of the immune response in disease progression. Notably, recent investigations highlight the significant involvement of NLRP3 inflammasomes in various autoimmune diseases, prompting an in‐depth exploration of their impact on MS.

**Method:**

The review focuses on elucidating the activation mechanism of NLRP3 inflammasomes within microglia/macrophages (MG/MФ), examining how this activation promotes an inflammatory response that exacerbates neuronal damage in MS. A comprehensive analysis of existing literature and research findings forms the basis for understanding the intricate interplay between NLRP3 inflammasomes and MS pathogenesis.

**Results:**

Synthesizing current research, the review provides insight into the pivotal role played by NLR inflammasomes, specifically NLRP3, in MS. Emphasis is placed on the inflammatory response orchestrated by activated MG/MФ, elucidating the cascade that perpetuates neuronal damage in the disease.

**Conclusions:**

This review concludes by consolidating key findings and offering a nuanced perspective on the role of NLRP3 inflammasomes in MS pathogenesis. The detailed exploration of the activation process within MG/MФ provides a foundation for understanding the disease's underlying mechanisms. Furthermore, the review sets the stage for potential therapeutic strategies targeting NLRP3 inflammasomes in the pursuit of MS treatment.

## Graphical abstract

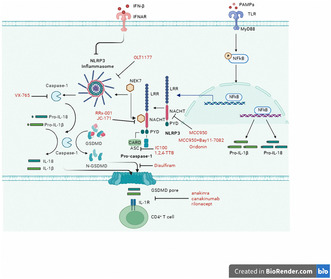

## 1 Introduction

**FIGURE 1:**
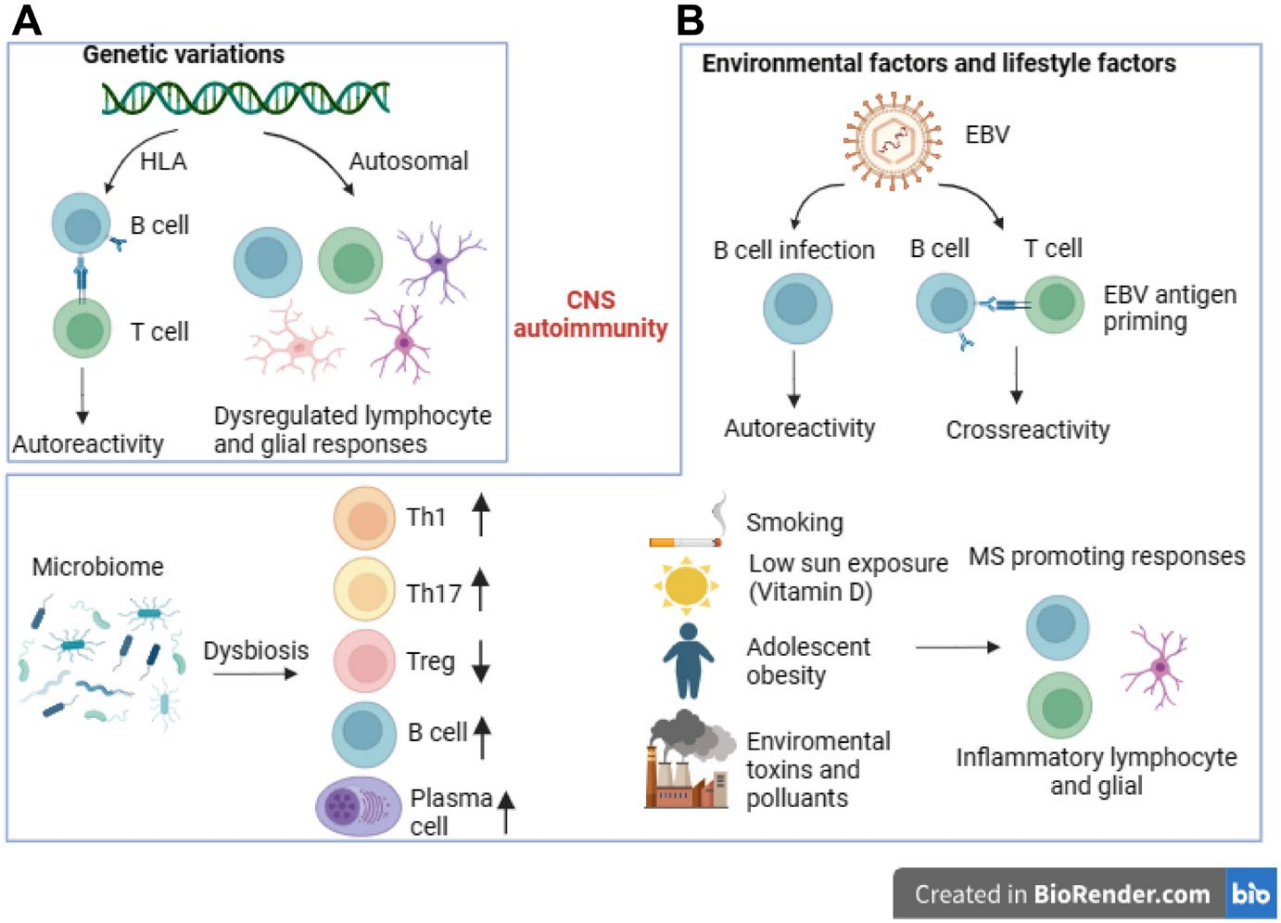
Genetic, environmental, and lifestyle factors influencing the onset of MS (created with BioRender). MS is the result of an autoimmune response caused by the dynamic interaction between genetic (A), environmental, and lifestyle factors (B).

Multiple sclerosis (MS) is a chronic central nervous system (CNS) autoimmune disease characterized by neuroinflammation and neurodegeneration, leading to a wide range of symptoms and complications, ultimately resulting in permanent neurological disability [1, 2]. According to MS Canada, the most common symptoms of MS include fatigue, bladder and bowel dysfunction, dizziness, cognitive and physical impairments, sexual dysfunction, optic neuritis, heat intolerance, and depression. MS is classified into several phenotypes based on clinical course: relapsing–remitting MS (RRMS), secondary progressive MS (SPMS), and primary progressive MS (PPMS), with approximately 80%–85% of patients being classified as RRMS [3]. While MS can occur at any age, it is most commonly diagnosed in young women and is a lifelong condition [1]. The global prevalence of MS has increased from 2.3 million in 2013 to 2.9 million by 2023, with one person being diagnosed with MS every five minutes, on average, at the age of 32 [3, 4]. MS is considered a global issue, with varying incidence and prevalence rates across different regions and significant disparities among different racial and ethnic groups, demonstrating a high degree of heterogeneity [5, 6, 7]. The regions with the highest reported MS prevalence are the Americas (117.49 per 100,000 people) and Europe (142.81 per 100,000 people), while Latin America has the lowest prevalence (ranging from 2.0 to 69.0 per 100,000 people). Asia shows substantial regional variation in MS prevalence, ranging from 3 to 50 cases per 100,000 people. Although the prevalence and incidence of MS in China are relatively low (with around 42,440 MS patients in 2020), considering the country's population of 1.4 billion, the total number of MS patients is significant [3, 8, 9]. The increasing prevalence of MS exacerbates the severity of the disease burden, leading to substantial social and economic consequences [10]. Despite the approval of over a dozen disease‐modifying therapies (DMTs) for MS treatment worldwide, with improvements in efficacy and choices, they remain primarily limited to the treatment of RRMS [2]. The etiology of MS, despite extensive research, remains incompletely understood. Evidence suggests that MS results from dynamic interactions between genetic, environmental, and lifestyle factors, leading to autoimmune reactions [2, 11] (Figure 1). Genetic studies have shown that the major genetic risk factors for MS are polymorphisms in the major histocompatibility complex (MHC) locus, specifically the HLA gene cluster. Although less extensively studied, autosomal and X‐chromosomal genes are also associated with MS susceptibility. Other environmental and lifestyle factors include the microbiome, Epstein–Barr virus infection, smoking, environmental exposures (sunlight and pollutants), and adolescent obesity [2]. Autoimmune reactions are considered the main mechanism driving the development of MS, with genetic, environmental, and lifestyle factors influencing individual susceptibility and disease progression. Notably, neuroinflammation plays a central role in the pathogenesis of MS. Inflammation, characterized by focal demyelinated regions in the CNS, can be observed at all stages of MS, including lesion sites and surrounding areas, often associated with activated MФ, MG, and T cells infiltration [12, 13]. NLR inflammasome activation also plays a crucial role in the autoimmune and pro‐inflammatory responses in MS [14]. Specifically, NLRP3 inflammasomes can influence the occurrence of neuroinflammation in MS through the regulation of MG, astrocytes, and CNS macrophage phenotypes [15, 16, 17]. Therefore, a deep understanding of the role of NLR inflammasomes and immune cells like MG and MФ in MS is of significant importance for unraveling the pathogenesis of MS and identifying new therapeutic targets.

## 2 Quantitative Study of the Literature Network Based on Citespace

**FIGURE 2:**
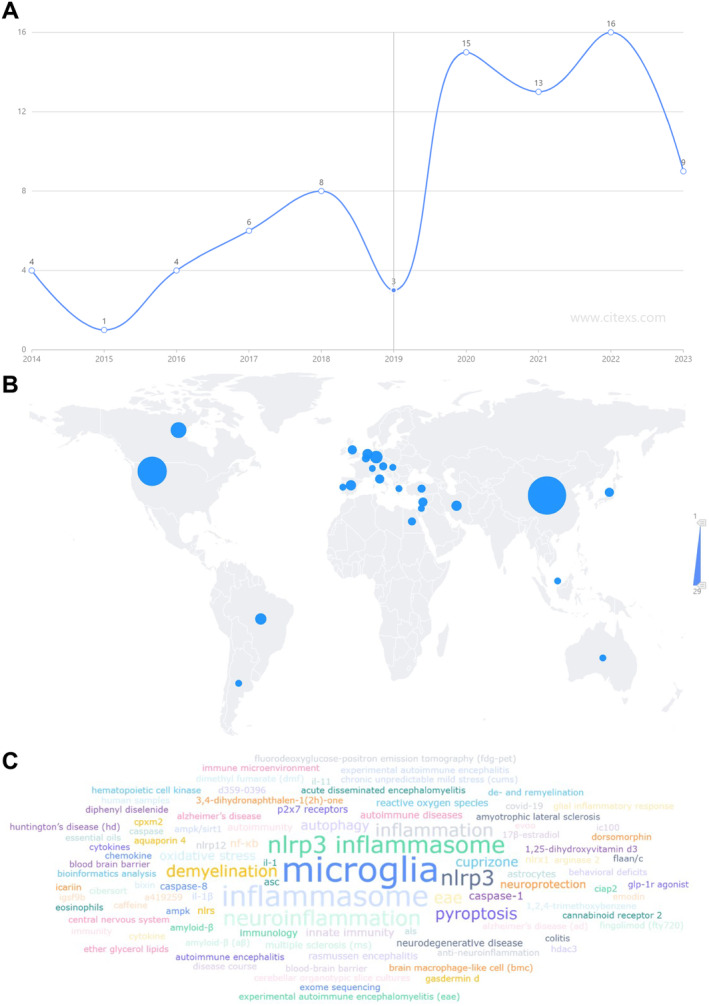
Trends in publication, affiliated countries, and hot topic frequency analysis of related literature. (A) In 2022, the number of publications peaked at 16 articles, and the fastest growth rate was observed in 2020, reaching 400%, indicating that research in this field is rapidly developing and in a phase of rapid growth. The *x*‐axis represents the year, and the *y*‐axis represents the number of publications, sourced from the PubMed database. (B) From January 2013 to October 2023, the top 24 countries worldwide in terms of publications in this research field are shown. China ranked first with 29 articles (36.71%), followed by the United States (21 articles, 26.58%), Canada (9 articles, 11.39%), and Italy (18 articles, 9.57%). The color scale on the right (1–29) represents the number of publications; larger circles in the figure indicate more publications, sourced from the CiteSpace data analysis platform. (C) Hot topic frequency analysis of the literature. The top five most frequently occurring keywords are MG, inflammasome, NLRP3 inflammasome, neuroinflammation, and NLRP3, sourced from the CiteSpace data analysis platform.

To comprehensively understand the role of NLR inflammasomes and MG/MФ in MS, we conducted a literature analysis and big data mining using the PubMed literature database and the CiteSpace data analysis platform. The research focused on MS/experimental autoimmune encephalomyelitis (EAE), as well as studies on inflammasomes/NLR/NLRP3 and MG/MФ from January 2013 to October 2023. We performed data mining and analysis on nationalities, keywords, and associated genes and visually displayed this field's overall trends, distribution patterns, and hotspots. Seventy‐nine relevant articles were retrieved, with an average of 8 publications annually. As shown in Figure 2A, there was a peak in the number of publications in 2022, reaching 16 articles, and the highest growth rate was in 2020, reaching 400%. These findings indicate that research in this field is rapidly developing and growing. From January 2013 to October 2023, the top 24 countries in terms of publications in this research field worldwide are shown in Figure 2B. China (29 articles, 36.71%) ranked first, followed by the United States (21 articles, 26.58%), Canada (9 articles, 11.39%), and Italy (18 articles, 9.57%).

**FIGURE 3:**
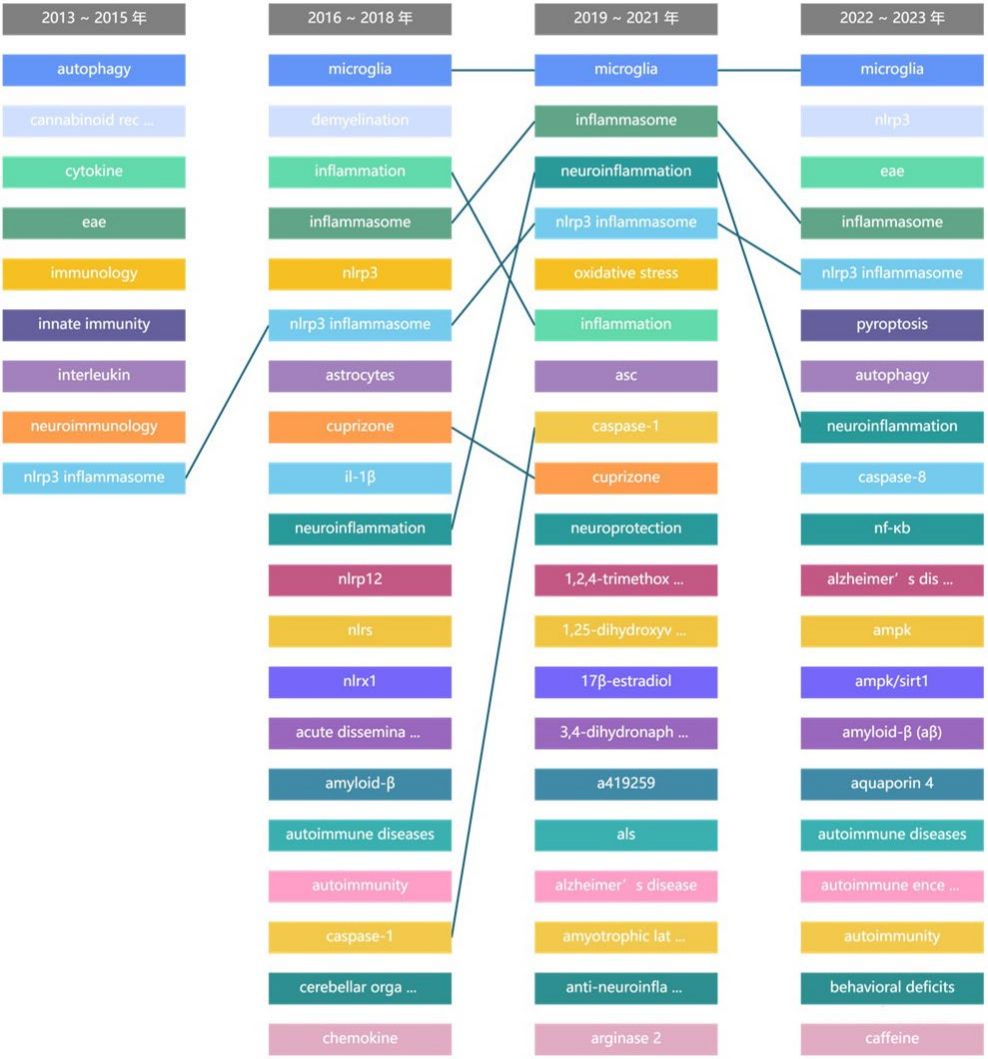
Ranking and changes in popularity of keywords over different time periods (sourced from CiteSpace Data Analysis Platform). Initially (2013–2015), the focus was on NLRP3 inflammation, gradually followed by keywords related to inflammasomes, neuroinflammation, and other inflammation‐related studies.

**FIGURE 4:**
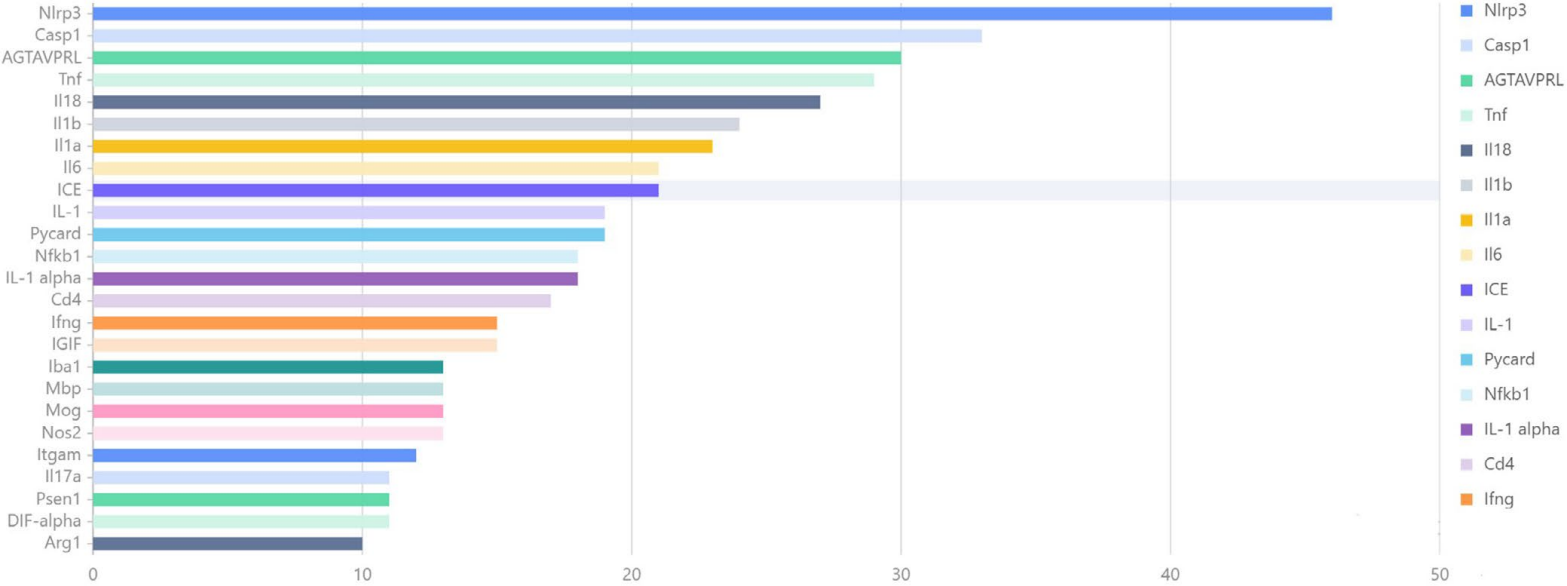
Analysis of literature affiliation by gene (sourced from BioBERT Biomedical Language Representation Model). The x‐axis represents the number of publications. The BioBERT biomedical language representation model was used to mine and statistically analyze gene entities in the abstracts of these 79 articles. The gene with the highest number of publications is Nlrp3 (46 articles), followed by Casp1 (33 articles), and AGTAVPRL ranked third (30 articles).

Keywords in a paper provide a concise and condensed summary of research objectives, research subjects, and research methods. An analysis based on keywords can reflect the evolving trends and research hotspots in a certain research field over a specific period. As shown in Figure 2C, the top 5 keywords with the highest occurrence frequency are MG, inflammasome, NLRP3 inflammasome, neuroinflammation, and NLRP3. The ranking and changes in the popularity of keywords in each period are displayed in Figure 3. Additionally, we used the BioBERT biomedical language representation model to mine and analyze the gene entity words in the abstracts of these 79 articles. As shown in Figure 4, the gene with the highest number of publications is Nlrp3 (46 articles), followed by Casp1 (33 articles), and AGTAVPRL ranked third (30 articles).

## 3 Pathology of MS

Among the clinical forms of MS, relapsing–remitting MS (RRMS) is the most common, with a diagnosis rate of 85%–90% among patients aged 20–40. RRMS is characterized by the occurrence (relapse) and recovery (remission) of symptoms. After an average of 10 to 15 years, the disease progresses to secondary progressive MS (SPMS), which is characterized by gradually worsening and irreversible neurological impairments. Approximately 10%–15% of patients, especially those diagnosed at an older age, present with a progressive disease course from the onset, known as primary progressive MS (PPMS) [2, 18].

In the early stages of RRMS, central nervous system lesions are called active lesions. These lesions are characterized by demyelination, myelin debris, infiltration of immune cells (particularly around blood vessels), blood–brain barrier disruption, reactive astrocytosis, and axonal damage. Active lesions are mainly associated with the early disease stage in RRMS patients [19]. Additionally, there are also mixed active/inactive lesions, which present with demyelination but exhibit activated small glial cells/MФ at the borders, foamy or bifurcated phagocytic cell phenotypes, axonal damage, subtle blood–brain barrier disruption, and infiltration of immune cells adjacent to most of the lesions [19]. Both active/inactive lesions are associated with MS progression and are more common in elderly patients [20].

The most specific pathological changes in MS are focal lesions accompanied by primary demyelination and astrocytic scar formation. These lesions occur against the backdrop of chronic inflammation and are not limited to focal lesions alone, being present in significant quantities in white matter, cortical gray matter, deep brainstem nuclei, and the spinal cord [18, 19]. Therefore, multiple regions of the central nervous system and tissue microenvironments contribute to the pathological process of MS, with their contributions varying depending on individual patients, disease forms, courses, or stages.

## 4 Role of NLR Inflammasomes in MS

### 4.1 Activation of NLR Inflammasomes

**FIGURE 5:**
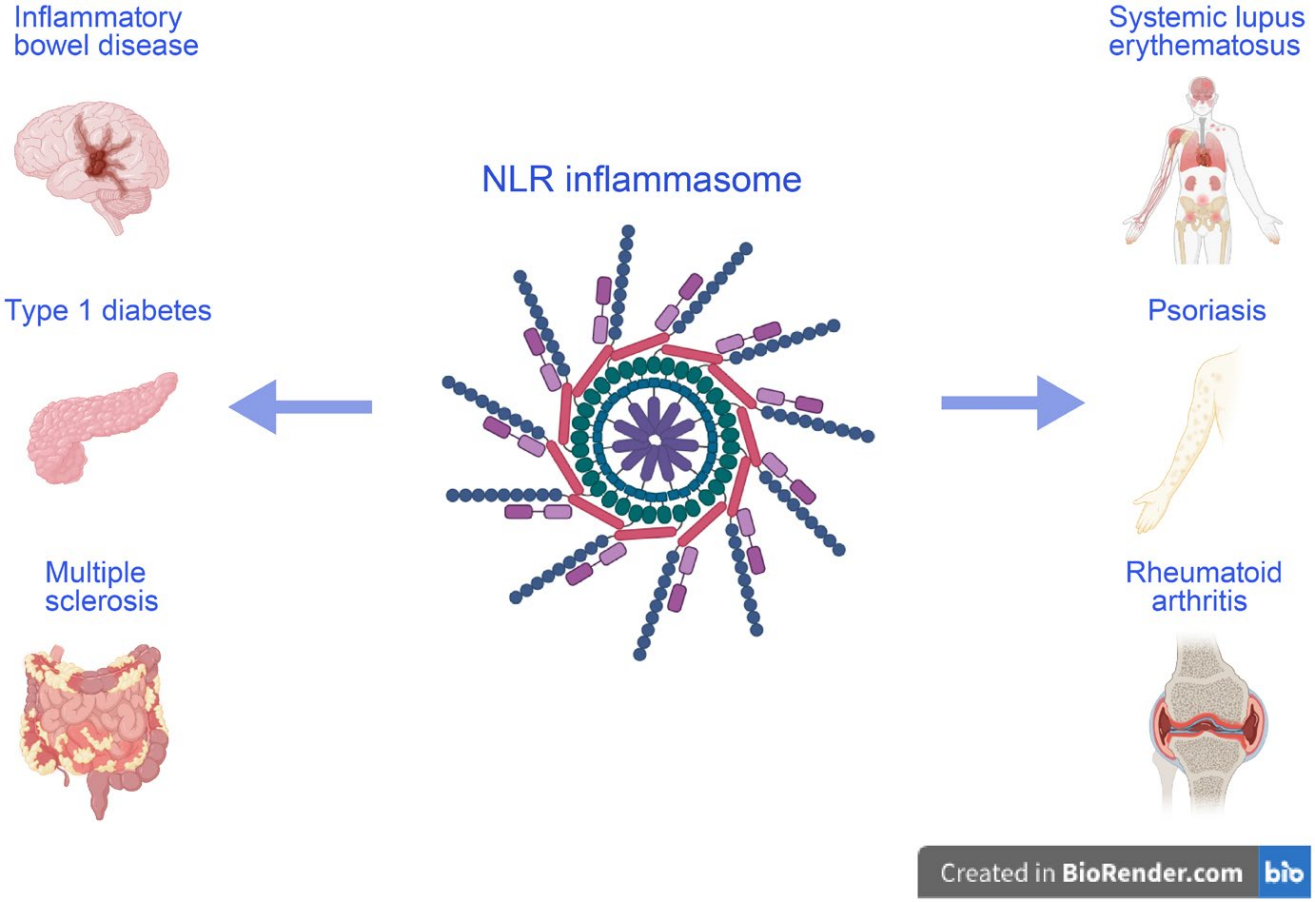
Involvement of NLRs in the regulation of autoimmune diseases (created with BioRender). Abnormal activation of NLR inflammasomes is one of the mechanisms underlying various autoimmune diseases, including inflammatory bowel disease, rheumatoid arthritis, MS, systemic lupus erythematosus, psoriasis, and type 1 diabetes.

Innate immunity serves as the primary defense system against microbial infections. Pattern recognition receptors (PRRs) are crucial in detecting invading pathogens [21]. The nucleotide‐binding oligomerization domain (NOD)‐like receptors (NLRs) constitute the major family of intracellular PRRs. They participate in combating pathogen invasion and contribute to maintaining normal physiological balance. Some NLRs form multiprotein complexes known as inflammasomes, which play a critical role in the innate immune response to DAMPs released by dying or stressed cells, as well as PAMPs released by bacteria or viruses [12]. Aberrant activation of NLR inflammasomes is one of the mechanisms underlying various autoimmune diseases [22] (Figure 5), such as inflammatory bowel disease (IBD) [23], rheumatoid arthritis (RA) [24], MS [25], systemic lupus erythematosus (SLE) [26], psoriasis [27], and type 1 diabetes (T1D) [28].

Since discovering the first inflammasome in 2002, several types have been identified, including NLRP1, NLRP3, NLRP6, and NLRC4 [29, 30]. The NLRP3 inflammasome is the largest and most well‐defined multiprotein complex observed to date. The innate immune sensor NLRP3, the adaptor molecule ASC, and the effector protein pro‐caspase‐1 [31]. The activation of NLRP3 involves two stages: priming and activation. During the priming stage, NLRP3 is activated by binding to PAMPs or DAMPs, triggering the activation of membrane‐bound TLRs, typically TLR4. This activation signal is then transduced into the cell, stimulating the nuclear factor (NF)‐κB pathway, which induces the expression of NLRP3, pro‐interleukin‐1β (IL‐1β), pro‐IL‐18, and pro‐caspase‐1. This step represents the first signal. The second signal comes from changes in the intracellular environment, such as potassium efflux, lysosome rupture, or reactive oxygen species (ROS) production. These alterations can directly or indirectly activate NLRP3. Once activated, the NLRP3 inflammasome activates pro‐caspase‐1 into its mature form, which further cleaves pro‐IL‐1β and pro‐IL‐18 into their mature forms (IL‐1β and IL‐18). Additionally, active caspase‐1 cleaves Gasdermin D (GSDMD) into an N‐terminal domain (GSDMD‐N) and a self‐inhibitory C‐terminal domain (GSDMD‐C). GSDMD‐N oligomerizes and binds to acidic phospholipids in the plasma membrane, forming pores that disrupt membrane integrity, leading to the release of IL‐1β and IL‐18 and inducing pyroptosis [12, 32]. Mitochondrial dysfunction plays a crucial role in the activation of the NLRP3 inflammasome. Stimulation of the NLRP3 inflammasome results in mitochondrial dysfunction, leading to the generation and release of mitochondrial reactive oxygen species (mtROS), the release of mitochondrial DNA (mtDNA) into the cytoplasm, and Ca^2+^ signaling, which further activates the NLRP3 inflammasome. Moreover, lysosome rupture triggers the release of lysosomal contents, activating the NLRP3 inflammasome [33].

**FIGURE 6:**
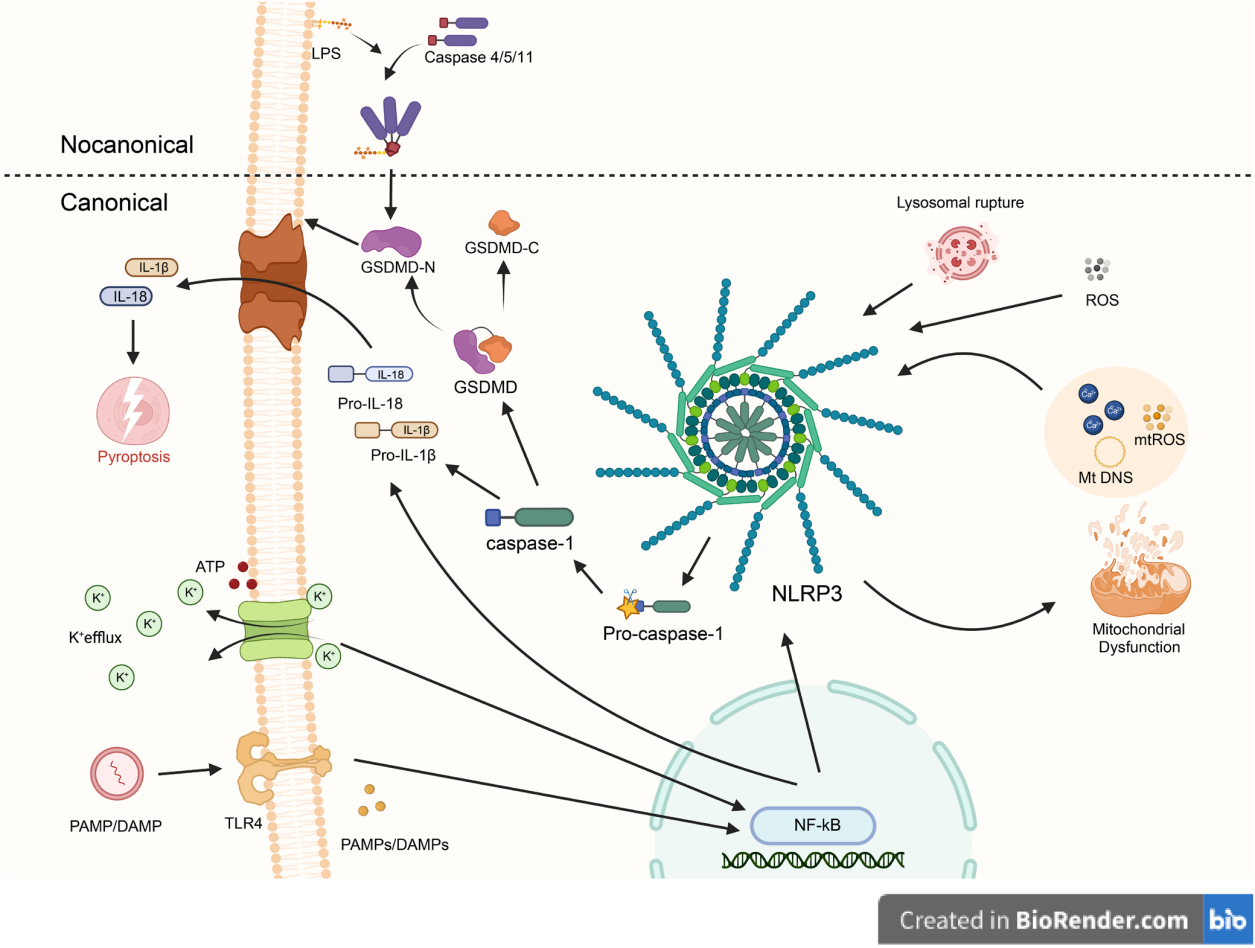
Activation mechanisms of NLRP3 inflammasomes [12] (created with BioRender). NLRP3 activation includes both classical and non‐classical pathways. Classical activation pathway: The priming stage of NLRP3 is initiated by binding to PAMPs or DAMPs, activating membrane‐bound TLRs, typically TLR4. The activation signal transduces into the cell, stimulating nuclear factor (NF)‐κB, which induces the expression of NLRP3, pro‐IL‐1β, pro‐IL‐18, and pro‐caspase‐1, representing the first signal. The second signal arises from changes in the intracellular environment, such as potassium efflux, lysosomal rupture, or the production of ROS. These changes can directly or indirectly activate NLRP3. Non‐classical activation pathway: Mediated by caspase‐11 in rodents and its human homologs caspase‐4/5. This means that in mice, Caspase‐11, and in humans, caspase‐4/5, can directly sense cytosolic lipopolysaccharide (LPS), leading to pore formation in the plasma membrane, activating GSDMD, inducing pyroptosis, and causing K^+^ efflux.

In addition to the classical activation pathway, the NLRP3 inflammasome can be activated through a non‐classical pathway mediated by caspase‐11 in rodents and its human homologs caspase‐4/5. It means that in mice, Caspase‐11, and humans, caspase‐4/5 can directly sense cytosolic lipopolysaccharide (LPS), resulting in the formation of pores in the plasma membrane, activating GSDMD, inducing pyroptosis, and promoting K+ efflux. This triggers a non‐classical activation pathway of the NLRP3 inflammasome, independent of caspase‐1 [12, 32] (Figure 6).

### 4.2 Genetic Variations of NLR Inflammasomes in MS Pathogenesis

Research has shown that the activation of inflammasomes plays a crucial role in both the autoimmune and pro‐inflammatory responses of MS [34]. A recent study conducted genetic and functional analyses of inflammasomes and identified rare homogeneous variants of NLRP1 (p.Ile601Phe and p.Ser1387Ile), a variant of NLRP3 (p.Leu832Ile), which substitutes an amino acid in a crucial leucine‐repeat domain, as well as variants of NLRC4 (p.Arg310Ter and p.Glu600Ter), leading to protein truncation in some MS patients. These findings suggest that rare protein variants may be associated with the activation of NLR receptors and may increase the risk of developing MS [35]. However, the exact mechanisms underlying the involvement of NLRP1 variants in MS pathogenesis remain unclear. A study by Maver et al. found a potential pathogenic amino acid substitution (Gly587Ser) in NLRP1, which was associated with increased production of pro‐inflammatory cytokines IL‐18 and IL‐1β in familial MS patients, as well as overall activation of the NLRP1‐mediated signaling pathway [36]. Nevertheless, another study failed to identify pathogenic genetic variations in the NLRP1 gene [37]. Genetic studies on NLRC4 variants indicate an association between loss‐of‐function variation (rs479333) and beneficial response to IFN‐β treatment, as well as lower levels of IL‐18 [38]. Recent research has also implicated variations in NLRP3‐related genes as being associated with susceptibility to MS [33]. Imani et al. analyzed the association between NLRP3 single nucleotide polymorphisms (SNPs) (rs‐10754558, rs‐35829419, rs‐3806265, rs‐4612666) and susceptibility to MS, highlighting the crucial role of polymorphisms in the development of MS [39]. Furthermore, a functional genetic variation (Q705K) in NLRP3 has been found to be correlated with the severity of MS [38]. These studies demonstrate a potential association between genetic variations in NLR receptors, such as NLRP1, NLRP3, and NLRC4, in inflammasomes and the development, severity, and treatment response of MS.

### 4.3 The NLRP3 Inflammasome and Its Related Factors Mediate the Occurrence of MS

In 2020, Malhotra et al. identified the NLRP3 inflammasome as a prognostic factor and therapeutic target for primary progressive MS (PPMS) [40, 41]. Through bioinformatics analysis, Li et al. determined that NLRP3, LILRB2, C1QB, CD86, C1QA, CSF1R, IL1B, and TLR2 are eight core genes associated with MS [42]. EAE is the most commonly used animal model for human MS [43]. In the relapsing–remitting EAE (RR‐EAE) mouse model, inhibiting NLRP3 inflammasome activation may be a potential therapeutic approach to alleviate central nervous system (CNS) pathological pain (CPP) and disease relapse in RR‐MS mice [44]. RRMS patients exhibit significant upregulation of NLRP3, ASC, and caspase‐1, as well as a decrease in serum TGF‐β1 and an increase in IL‐1β levels [45]. MS is typically triggered during infection, with Epstein–Barr virus (EBV) infection being associated with MS. Recent analyses of a large collection of prediagnostic blood samples showed that the risk of developing MS increased 32‐fold after EBV infection, while the risk did not increase after infection with other viruses [46]. EBV replication activates the NLRP3 inflammasome, leading to virus clearance and induction of cell pyroptosis [47]. Studies report an increased expression of the NLRP3 and IL‐1β genes in the brains and cerebrospinal fluid (CSF) of MS patients, as well as elevated levels of ASC, caspase‐1, and IL‐18 in serum [48, 49]. Nlrp3 expression is upregulated in the spinal cord during EAE, and Nlrp3−/− mice exhibit significantly delayed disease progression and reduced severity. Nlrp3−/− mice with EAE show decreased IL‐18 production, similar to IL‐18−/− mice [50]. Recently, it was reported that thymic stromal lymphopoietin (TSLP) directly induces NLRP3 expression through Janus kinase (JAK)2 phosphorylation. Tslpr−/− mice show decreased JAK2 phosphorylation and NLRP3 expression in the brain, as well as significantly reduced EAE scores [51]. As a newly discovered pyroptotic factor downstream of the NLRP3 inflammasome, GSDMD is essential in the pathogenesis of EAE. The lack of GSDMD in peripheral myeloid cells impairs immune cell infiltration into the CNS, thus inhibiting neuroinflammation and demyelination [52]. The NLRP3 inflammasome and its related factors play a crucial role in the etiology, disease progression, and treatment of MS, interacting with EBV infection and other molecular pathways.

## 5 Involvement of Glial Cells and MФ in the Pathogenesis of MS

### 5.1 Overview of the Role of Innate and Adaptive Immune Cells in the Pathogenesis of MS

**FIGURE 7:**
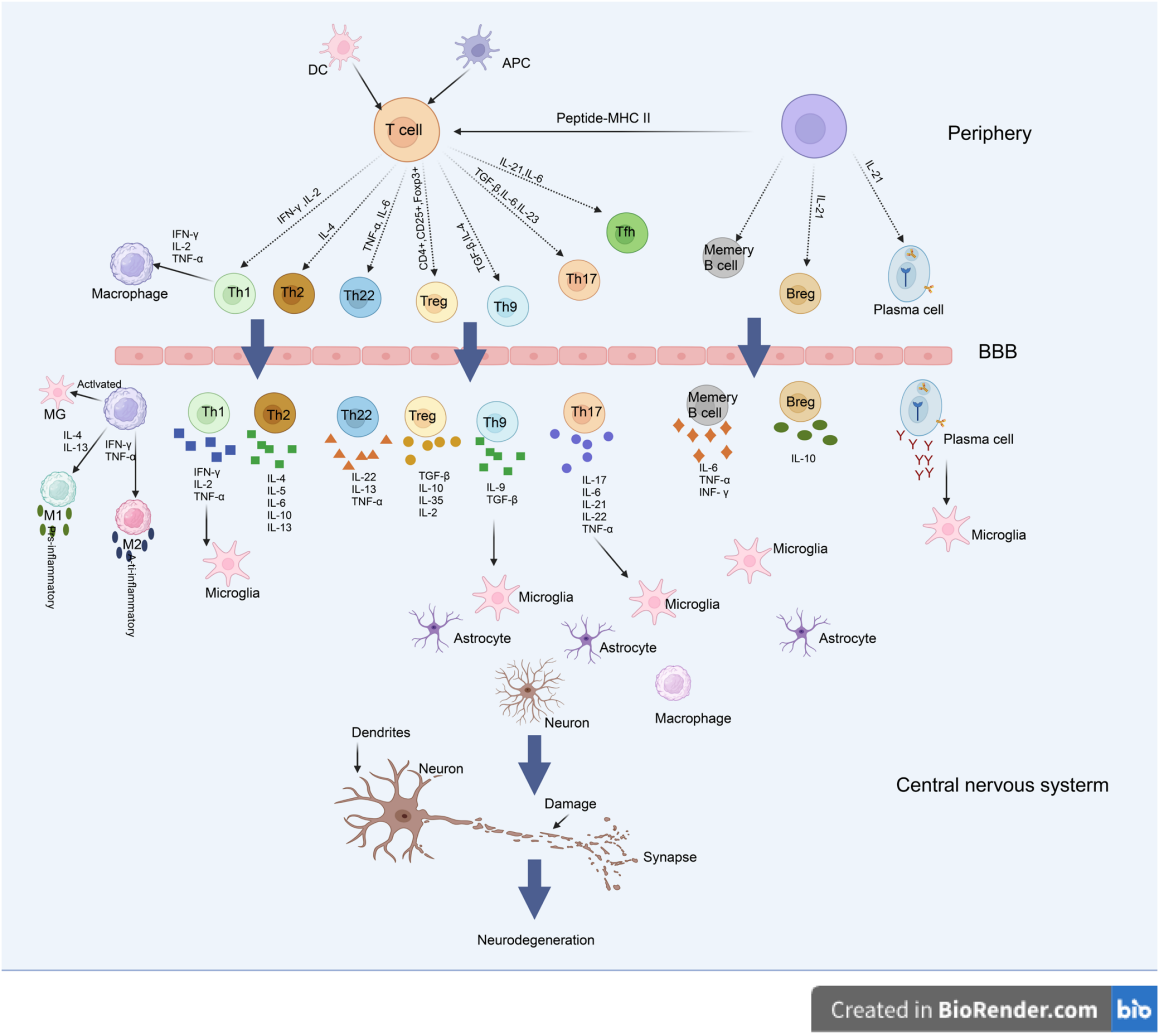
Roles of different immune cells in the pathogenesis of MS [55] (created with BioRender).

The central nervous system (CNS) inflammation in MS patients is caused by the infiltration of various immune cells. MS and EAE share the characteristic of immune cell trafficking across the blood–brain barrier (BBB) into the CNS, where interactions occur among different subsets of immune cells. This process is coordinated by a series of secreted molecules and surface molecules, including cytokines and chemokines [53]. Innate and adaptive immune cells, such as T cells, B cells, activated MΦs, MG, and dendritic cells (DCs), have been reported to participate in the pathogenesis of MS, leading to CNS inflammation, neurodegeneration, and demyelination [54, 55] (Figure 7).

MS and EAE are believed to be primarily driven by T lymphocytes in various immune cell populations [56]. T cells can be classified into subtypes based on different criteria. They can be divided into CD4^+^ T cells and CD8^+^ T cells based on the expression of CD4 or CD8 and into helper T cells (Th) and regulatory T cells (Treg) based on their function [57]. Th1 cells mediate cytotoxicity, activate MФ to enter the central nervous system, stimulate astrocytes, and cause myelin sheath damage, thus promoting inflammation. Th2 cells, on the other hand, exert a negative feedback effect on the proliferation and differentiation of Th1 cells, play an anti‐inflammatory role, and can protect against MS [58, 59]. It has been found that Th17 cells and IL‐17 levels are elevated in EAE mice, and the risk of EAE infection is significantly reduced when Th17 cells are decreased or when IL‐6 and IL‐23 are lacking [60, 61]. In addition, Th17 cells activate astrocytes and MФ to form lymphoid‐like follicular structures, which play important roles in acute and chronic MS [55]. The proportion of Th22 cells and IL‐22 in the serum of MS patients increases, particularly during disease activity, and is positively correlated with disease duration [62, 63]. The development of the EAE mouse model mediated by CD8^+^ T lymphocytes has demonstrated the pathogenic role of CD8^+^ T lymphocytes [64]. Treg cells have been described as a population that inhibits the function of pro‐inflammatory cells, and the transfer of Treg cells can reduce the severity of EAE [65]. It has been found that Tfh cells infiltrate the CNS of EAE mice, and treatment with anti‐Tfh chemokine ligands significantly reduces the incidence of EAE disease [66].

In the past, MS was believed to be a T lymphocyte‐mediated autoimmune disease. However, recent studies have shown excellent clinical efficacy of B cell therapy in treating MS, suggesting that B cells may play a more central role in the pathogenesis of MS [55, 67]. B cells mediate central nervous system damage through various mechanisms, including antigen presentation, release of autoreactive antibodies, secretion of pro‐inflammatory cytokines, and formation of ectopic lymphoid tissue [55]. The efficacy of anti‐CD20 therapy in eliminating B cells has also demonstrated the key role of B lymphocytes in neuroinflammation in EAE and in relieving relapsing–remitting MS [68].

In addition to these major subtypes of immune cells, some innate immune cells also play critical roles in the pathogenesis of MS. MФ in the central nervous system lesions of MS patients are usually divided into M1 and M2 subtypes [69]. M1 cells are more likely to induce tissue damage by secreting pro‐inflammatory molecules, leading to the differentiation of T cells into Th1 and Th17 cells. In contrast, M2 cells have a protective effect and promote tissue repair by secreting anti‐inflammatory cytokines and producing extracellular matrix molecules [70]. Tissue‐resident MФ in the brain parenchyma, also known as MG and monocyte‐derived MФ, play similar roles. MG are resident innate immune cells in the central nervous system. When activated by external stimuli, they secrete pro‐inflammatory and chemotactic factors, recruiting MФ and T cells, among other inflammatory cells, to the central nervous system. MФ also secrete pro‐inflammatory and chemotactic factors, further promoting inflammation progression and inflammatory cell infiltration. Activated MG also releases matrix metalloproteinases, which disrupt the blood–brain barrier and facilitate inflammatory cell infiltration [55]. MG, like MФ, can change their phenotype under different conditions and environmental factors, transitioning between pro‐inflammatory and anti‐inflammatory phenotypes. Conventional dendritic cells (cDC) and plasmacytoid dendritic cells (pDC) are significantly increased in the CSF of MS patients. In EAE, cDC activates CD4^+^ T cells to differentiate into Th1 and Th17 cells, while pDC promotes Treg differentiation. Studies have shown that dendritic cells may have opposing pro‐inflammatory and anti‐inflammatory functions in EAE and MS, which mainly depend on the stage, form, and type of dendritic cells [56, 71].

In summary, the pathogenesis of MS involves complex interactions among various immune cells. A deeper understanding of these cells and their functions can contribute to the development of more effective treatment strategies.

### 5.2 The Mechanism of Action of MG and MФ in MS

Both MG and MФ exhibit similarities in certain functions and play crucial roles in the initiation, maintenance, and resolution of inflammation, thereby exacerbating damage to the central nervous system (CNS). Furthermore, these cells also have a central role in the disruption of the blood–brain barrier (BBB). This similarity prompts further exploration of the interaction between MG and MФ in MS and their potential therapeutic implications.

**FIGURE 8:**
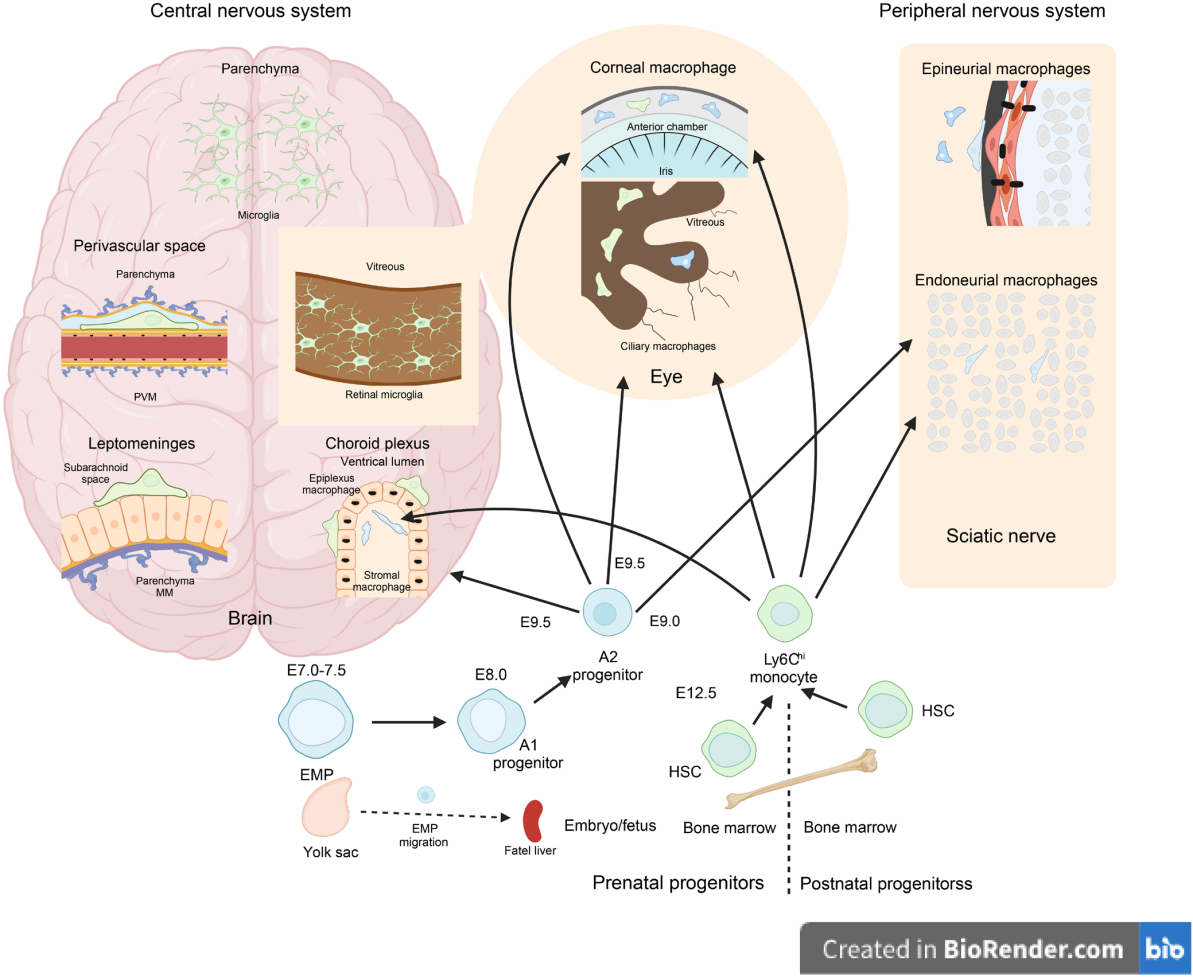
Anatomy and origins of MG and CNS MФ [74] (created with BioRender). EMP, erythromyeloid progenitor cells; HSC, hematopoietic stem cells; MM, meningeal macrophages; PVM, perivascular macrophages.

MФ are innate immune phagocytic cells that detect PAMPs and DAMPs, expressed by pathogens and apoptotic cells, respectively. Additionally, MФ functions as antigen‐presenting cells (APCs) in adaptive immunity by presenting antigens to T lymphocytes [72]. Based on in vitro characteristics, MФ can be divided into M1 and M2 phenotypes. Exposure of MФ to Th1 cytokines, lipopolysaccharides (LPS), and granulocyte‐macrophage colony‐stimulating factor (GM‐CSF) induces polarization towards a pro‐inflammatory M1 phenotype. Conversely, exposure of MФ to Th2 cytokines and other immunoregulatory agents, including macrophage colony‐stimulating factor (M‐CSF), IL‐10, transforming growth factor (TGF)‐β, and vitamin D3, leads to polarization towards an anti‐inflammatory M2 phenotype [72]. The normal adult brain contains four types of resident mononuclear phagocytes collectively known as CNS‐associated macrophages (CNS MФ). CNS MФ includes MG located throughout the brain parenchyma and three types of CNS border‐associated macrophages (BAM), which are situated between the CNS and BBB. BAM consists of perivascular macrophages (PVM), meningeal macrophages (MM), and choroid plexus MФ [73, 74]. Despite anatomical differences, MG and BAM share similarities in their development, as they both originate primarily from embryonic progenitor cells in the yolk sac, which migrate and mature in the brain (Figure 8).

In the central nervous system (CNS), the role of MG is well‐established. Through genome‐wide association studies (GWAS), Ma et al. successfully identified MS susceptibility variants, demonstrating significant enrichment of MS‐GWAS associations in the regulatory regions of MG and peripheral immune cell subtypes, particularly B cells and monocytes, but not in other brain cell types [75]. A previous study indicated that MS risk genes are significantly enriched in MG within the CNS [76]. A single‐cell RNA sequencing (scRNA‐Seq) study revealed that MG, oligodendrocyte precursor cells, endothelial cells, and astrocytes belong to the resident cell types in the CNS, exhibiting the greatest transcriptional modifications during EAE [53]. These three studies provide direct genetic evidence for the involvement of MG in MS susceptibility.

Under normal surveillance conditions in a healthy CNS, MG possesses small cell bodies with complex and highly branched morphologies [77]. In neuroinflammatory environments like MS, MG becomes activated, leading to transcriptional, biochemical, and metabolic remodeling to adopt new inflammatory functions [78, 79, 80]. Reactive MG undergo expansion and develop rounder cell bodies, exhibiting simpler branching patterns with shorter and thicker cellular processes. Reactive MG involves the downregulation of numerous homeostatic genes, indicating impaired and lost key homeostatic functions during MS that may exacerbate neurodegenerative processes in progressive MS pathology [78, 81, 82, 83]. For example, the regulating factor TREM2, involved in phagocytosis and chemotaxis, is highly expressed in myelin‐laden phagocytes at MS lesion sites and is an important defensive response factor against inflammation and injury in MS. TREM2 agonists promote myelin clearance and enhance remyelination in a demyelination animal model [83, 84]. A lipid metabolism‐related gene associated with TREM2 is apoE, which is upregulated in MG from EAE mice and associated with disease progression. Activation of the TREM2‐APOE pathway impairs MG homeostatic regulation in EAE [78]. Cx3cr1, another homeostatic gene, encodes the fractalkine receptor on MG involved in synaptic pruning and remodeling, which is also lost in EAE [78]. While reactive MG and MФ with more pro‐inflammatory phenotypes may contribute significantly to neurodegenerative processes during MS, the failure of MG and MФ to maintain repair and homeostatic functions is also crucial for the continuation of neurodegeneration in progressive stages of the disease.

**FIGURE 9:**
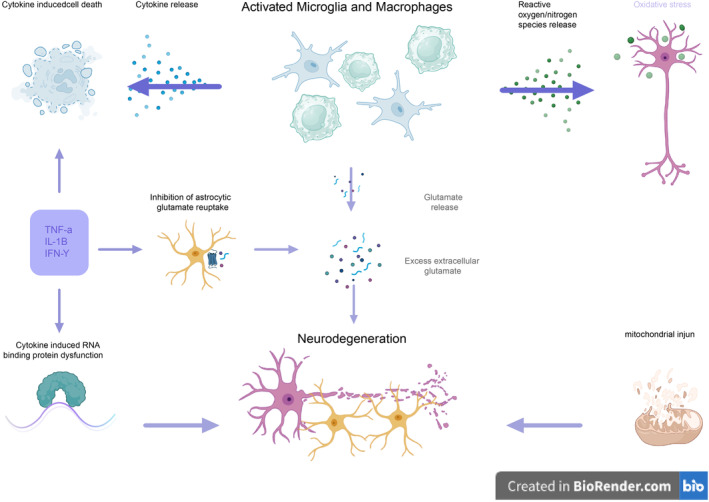
Mechanisms of neurodegeneration mediated by MG and MФ in progressive MS [77] (created with BioRender).

Pathological features of MS include iron deposition, blood–brain barrier (BBB) leakage, and meningeal inflammation, which may mediate the reactivity of MG and MФ, as well as neurodegeneration in MS [77]. In certain cases, MG and MФ may also contribute to progressive neurodegeneration in MS through the release of neurotoxic factors such as reactive oxygen species and nitrogen species (ROS/RNS), glutamate, and inflammatory cytokines IL‐1β, IL‐6, and tumor necrosis factor alpha (TNF‐α), leading to demyelination, neuronal loss, and axonal and mitochondrial damage in lesions [77] (Figure 9).

In MS, there is a phenotypic transformation of MФ and infiltration of MG, which can be divided into inflammatory M1 type and anti‐inflammatory M2 type. The M1/M2 ratio is an important factor influencing the recurrence of EAE. In the early stages of MS and EAE, MG recruited MФ to exhibit a pro‐inflammatory M1 phenotype. M2 MG plays a crucial role in recruiting and differentiating oligodendrocyte precursor cells (OPC) through the clearance of myelin debris. The anti‐inflammatory cytokines produced by M2 cells, such as IL‐4, IL‐10, and IL‐13, as well as activin A, are involved in the differentiation of OPC during remyelination [72, 85].

MФ contributes to the progression of EAE paralysis and demyelination by expressing MHC‐II, co‐stimulatory molecules, and producing pro‐inflammatory factors [86]. Both M1 and M2 MФ can be detected in MS lesions, with the expression of typical M1 markers higher than M2 markers in active and chronically active MS lesions [87]. During MS, M1 MФ secrete high levels of pro‐inflammatory cytokines, reactive oxygen, and RNS, as well as chemoattractants CCL4, CCL5, CCL8, CXCL9, CXCL10, and CXCL2, which recruit immune cells and exacerbate neuroinflammation and tissue damage [88]. In the deteriorating phase of the disease in mice, there is a higher level of M1 MФ infiltration in the central nervous system, while the gradual increase of M2 MФ is associated with improved neuronal function [89].

M2 MФ contributes to an anti‐inflammatory state and tissue repair by secreting cytokines such as IL‐4, IL‐10, IL‐13, and TGF‐β. These cells also promote the recruitment and differentiation of Th2 and Treg cells, inhibiting the inflammatory response in EAE mice [90]. M2 MФ expresses scavenger receptors to clear myelin debris in the damaged spinal cord, promoting central nervous system repair [91].

However, in MS, the phenotypic transformation of MG and MФ is not absolute. Some studies have found that they may simultaneously express markers of both M1 and M2 types, indicating the possibility of having an intermediate phenotype with both pro‐inflammatory and anti‐inflammatory functions [87]. The phenotype and function of MG and MФ appear to be influenced by environmental conditions [92]. Overall, the phenotypic transformation of MG and MФ in MS is a complex and dynamic process influenced by various factors. Targeting the imbalance between the pro‐inflammatory and anti‐inflammatory functions of MG and MФ may be a therapeutic option to promote remyelination in MS.

## 6 Activation of the NLRP3 Inflammasome in MG and MФ Mediates the Occurrence of MS

Studies have reported that inflammasome activation, especially in MG and MФ, is associated with aging and age‐related neurodegenerative diseases such as Alzheimer's disease, Parkinson's disease, Huntington's disease, MS, adenoviral diseases, and amyotrophic lateral sclerosis [93]. According to existing research, MG, astrocytes, and CD4^+^ T cells are the main cell types involved in NLRP3 inflammasome activation in MS. Studies have shown a positive correlation between the proportion of MG expressing NLRP3 and IL‐1β and the degree of demyelination in MS patients [40]. Furthermore, activation of NLRP3 inflammasome in MG converts astrocytes into a neurotoxic A1 phenotype, exacerbating cognitive deficits in MS [16]. There is substantial evidence indicating that NLRP3 inflammasome activation in MG contributes to MS pathogenesis and progression by recruiting activated T cells to the central nervous system (CNS) and promoting their cytokine release, leading to increased inflammation [94]. Zhang et al. reported the mechanism of atypical NLRP3 inflammasome activation in MG during the effector phase of EAE, suggesting that the deficiency in the adaptor molecule ASC of NLRP3 inflammasome in MG may weaken T cell proliferation and neutrophil infiltration. Caspase‐8 is activated in MG in an NLRP3/ASC‐dependent manner and plays a crucial role in the development of MS and CNS inflammation in EAE mice [95]. Another study showed that TRPV1 mediates NLRP3 inflammasome activation in MG, and TRPV1 deficiency alleviates EAE in mice by inhibiting NLRP3 inflammasome activation and reducing neuroinflammation [96]. Wang et al. found that IRAK‐M negatively regulates NLRP3 inflammasome in MG by inhibiting IRAK1 phosphorylation, thereby alleviating MG inflammation in EAE [97]. Inhibition of the NLRP3 inflammasome pathway has been shown to suppress in vitro activation of MG by inhibiting M1 polarization. The potential neuroprotective effect of nebivolol in a mouse model of MS is partly attributed to the shift of MG towards an M2 phenotype, alleviation of NLRP3 inflammasome activation, and reduction of oxidative stress [98]. In the early stages of EAE, activated caspase‐8 associated with NLRP3 aggregates was found in infiltrating monocytes‐derived MФ within demyelinated lesions. Caspase‐8 is partially activated in MФ and MG in active MS lesions, and conditional deletion of caspase‐8 in bone marrow cells exacerbates neuroinflammation and clinical symptoms of EAE [99]. Dimethyl fumarate, an approved MS treatment, exerts its therapeutic effects by inhibiting NLRP3 activation in MФ [100].

A study has shown that in animal models of MS, the activation of inflammasomes in bone marrow cells (MФ and MG) mediated by GSDMD drives the mechanisms of neuroinflammation and demyelination [101]. Mice lacking A20 expression in MG are highly susceptible to the development of inflammatory central nervous system (CNS) pathology. Mechanistically, this heightened inflammatory state is caused by excessive activation of Nlrp3 inflammasomes in A20‐deficient MG, while A20 can inhibit Nlrp3 inflammasome activation and neuroinflammation in MG [49]. Further research has indicated that the expression of A20 in MG controls Nlrp3 inflammasome activation and CNS inflammation in the EAE mouse model of MS, and the absence of A20 expression in nonessential CNS MФ may also contribute to increased EAE pathology [49, 102]. Recently, it has been discovered that MФ and MG stimulated by inflammation exhibit decreased levels of NLRP3‐dependent peroxisomal proteins, and the peroxisomal damage in CNS MФ leads to neuroinflammation and demyelination in MS [103]. NLRP3 inflammasome components and microRNA‐223‐3p are upregulated in activated MФ and MG at sites of myelin damage, and microRNA‐223‐3p and the small molecule NLRP3 inhibitor MCC950 can inhibit inflammasome activation in MФ and MG in vitro, with MФ being more prone to inflammasome activation compared to MG in vitro [104]. The long non‐coding RNA (lncRNA) GAS5 has been identified as an epigenetic regulator of MG polarization, as it suppresses MG M2 polarization. Interfering with GAS5 in transplanted MG inhibits EAE progression and promotes remyelination [105]. Knockdown of lincRNA‐Cox2 promotes resting MG (CD11b + CD45med) and inhibits IL‐1β secretion, while silencing lincRNA‐Cox2 inhibits NLRP3 inflammasome activation and promotes autophagy in BMDM and MG [106]. FLaAN/C prevents cell pyroptosis by inhibiting the ROS/NF‐κB/NLRP3 signaling pathway and promotes the polarization of MФ and MG from the inflammatory M1 phenotype to the anti‐inflammatory M2 phenotype, significantly alleviating inflammation in the EAE mouse model [17].

These studies collectively demonstrate the crucial role of NLRP3 in the activation of MФ and MG in MS, which regulates their activation and phenotypic conversion, influencing the progression of neuroinflammation and demyelination in MS and EAE models. Various mechanisms and pathways, such as caspase‐8, TRPV1, and IRAK‐M, have been found to affect the activation of NLRP3 inflammasomes in MG and MФ, playing critical roles in the development of MS and CNS inflammation. Additionally, a series of studies have identified other key molecules, such as GSDMD, A20, and microRNA‐223‐3p, involved in the regulation of inflammasome activation and neuroinflammation in MФ and MG. In conclusion, these findings provide a deeper understanding of the activation of NLRP3 inflammasomes in MG and MФ and offer valuable insight for the development of novel MS therapeutic strategies.

## 7 Targeting NLR Inflammasome as a Potential Therapeutic Approach for MS

### 7.1 Targeted Therapies Against NLR Inflammasome for MS Treatment

**FIGURE 10:**
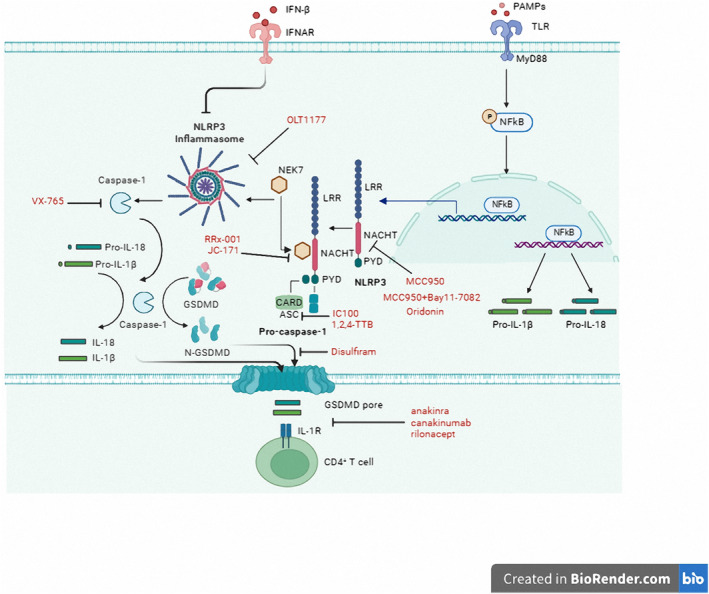
Inhibitors of the NLRP3 inflammasome activation pathway for the treatment of MS (created with BioRender).

Considering the pathological role of NLRP3 inflammasome in MS and EAE, it emerges as a promising therapeutic target for MS. Indeed, several therapies have recently been developed, targeting both the upstream and downstream pathways of NLRP3 inflammasome, including IFNβ treatment, inhibitors of upstream and downstream NLRP3 inflammasome activation pathways, and inhibitors specifically targeting NLRP3 inflammasome (Figure 10). The efficacy of these therapies has been validated in EAE models or clinical trials [32, 33] (Table 1).

**TABLE 1:** Clinical studies targeting NLRP3 and its related pathways.

No.	Description	References
Malhotra S. etc.	NLRP3 inflammasome and its related cytokine IL1B in the response to interferon beta in patients with relapsing–remitting multiple sclerosis	PMID: 25586466
Noroozi S. etc.	NLRP3, NLRC4, and AIM2 play critical roles in the progression of MS, probably by mediating Th1 and Th17 responses. It seems that decreased expression of IL‐1β is related to decreased production and also functions of inflammasomes	PMID: 27032392
Ozdogan H. etc.	Blocking IL‐1 is a safe and effective alternative for colchicine‐resistant FMF and probably also for associated MS	PMID: 32645635
NCT00731692	The anti‐inflammatory effects of fingolimod did not slow disease progression in primary progressive multiple sclerosis. Therapeutic strategies for primary progressive multiple sclerosis might need different approaches to those used for relapse‐onset multiple sclerosis	PMID: 26827074
NCT01194570	Among patients with primary progressive multiple sclerosis, ocrelizumab was associated with lower rates of clinical and MRI progression than placebo	PMID: 28002688
NCT03161028	In progress	
NCT03387670	In progress	
NCT02784210	In progress	

IFN‐β is one of the first‐line treatment options for MS, and it can inhibit the formation of NLRP1 and NLRP3 inflammasomes [12]. In a clinical study, 97 RRMS patients receiving IFN‐β therapy were categorized as responders or non‐responders based on clinical criteria after 24 months of treatment and radiological criteria after 12 months of treatment. The study found that in responders, the transcription levels of NLRP3 and IL‐1β in peripheral blood mononuclear cells significantly decreased, whereas in non‐responders, the expression of these markers increased after 3 months of treatment [107]. These results suggest that NLRP3 inflammasomes and IL‐1β play a role in the response of RRMS patients to IFN‐β. IFN‐β has been shown to effectively slow down the progression of EAE, but it is usually ineffective in NLRP3‐independent EAE [108]. Therefore, IFN‐β treatment for EAE and MS is dependent on NLRP3 inflammasomes. IFN‐β‐1a inhibits the production of pro‐IL‐1β upstream of NLRP3 inflammasomes by promoting the secretion of the immunosuppressive cytokine IL‐10 [109]. In peripheral blood mononuclear cells of MS patients receiving IFN‐β therapy, there is a significant decrease in NLRP3 mRNA expression and circulating levels of IL‐1β [110].

According to reports, β‐hydroxybutyrate can attenuate K+ efflux and inhibit the oligomerization of NLRP3 and ASC, thereby inhibiting the activation of NLRP3 inflammasomes [111]. Membrane‐associated protein 1 (Panx1) and P2X7R mediate K+ efflux, and the Panx1‐dependent mechanism (ATP release and/or inflammasome activation) promotes the progression of mouse EAE. Probenecid, a drug that blocks Panx1, weakens NLRP3 inflammasome activation, thereby alleviating neural inflammation in EAE mice [112, 113, 114]. Yu et al. found that Bixin, a carotenoid isolated from Bixa orellana seeds, can clear ROS through the NRF2 signaling pathway, inhibit MG aggregation, and suppress TXNIP/NLRP3 inflammasome activity, thereby reducing the severity of EAE [115]. In the early stages of MS, mitochondrial ROS stimulate NLRP3 inflammasomes, and some antioxidants targeting mitochondria have shown anti‐inflammatory and neuroprotective activities in animal models of MS [116]. Recent research suggests that curcumin‐loaded nanoparticles can inhibit the release of ROS and tissue protease B to hinder the infiltration of inflammatory monocytes through the blood–brain barrier and the proliferation of MG in EAE mice, thus inhibiting the progression of EAE [117]. In addition to these main upstream activation signals, NLRP3 inflammasome activation is also involved in other regulatory mechanisms. A recent study found that caffeine can reduce the activation of NLRP3 inflammasomes by inducing autophagy, thus alleviating the severity of EAE [118]. Another study found that CD47‐Fc fusion protein can reduce the production of NLRP3 inflammasome‐induced IL‐1β by promoting the production of NO, which contributes to the treatment of EAE [119].

IL‐1β and IL‐18 are major effector molecules of activated NLRP3 inflammasome. IL‐1β activates intracellular MyD88 and downstream signaling pathways, including NF‐κB and MAPK, ultimately triggering the activation of NLRP3 inflammasome [32]. IL‐1β contributes to the progression of MS [33]. Current therapeutic approaches studied include IL‐1β‐specific neutralizing antibodies (canakinumab), IL‐1 receptor antagonists (anakinra), and soluble decoy receptors for IL‐1α and IL‐1β (rilonacept) [120]. In a clinical trial, two MS patients treated with a combination of anakinra and canakinumab experienced significant alleviation of symptoms at 17 months and 6 years, respectively [121]. Anakinra exhibits good safety but has a short half‐life of only 4–6 h, necessitating daily injections [120]. On the other hand, canakinumab has better pharmacokinetics with a half‐life of 26 days, making it a preferable choice for MS treatment [121]. However, IL‐1β‐targeted therapy still has some limitations, including local inflammation, poor patient compliance due to frequent injections, and an increased risk of infection. Small molecule NLRP3 inhibitors may serve as suitable alternatives due to potentially higher efficacy and lower toxicity.

MCC950 is an effective and selective NLRP3 inhibitor that can bind to the Walker B motif of the NACHT domain of NLRP3 and inhibit ATPase activity, thereby alleviating the severity of EAE [122]. Bay11‐7082 has also been reported to inhibit the ATPase activity of NLRP3 and improve EAE, acting as a direct inhibitor of NLRP3 inflammasome [123, 124]. A follow‐up study investigated the combination of rapamycin and MCC950 and found that MCC950 enhanced the activity of rapamycin, resulting in improved therapeutic efficacy against MS [125]. JC‐171, a hydroxamic acid derivative, has been shown to target the NLRP3/ASC interaction stimulated by LPS/ATP, inhibit NLRP3 inflammasome activity and IL‐1β secretion, and attenuate EAE progression [126]. Oroxylin A, the major active component of traditional Chinese medicine Scutellariae radix, was demonstrated by He et al. to be a specific covalent inhibitor of NLRP3 inflammasome. Oroxylin A forms a covalent bond with cysteine 279 in the NACHT domain of NLRP3, disrupting the interaction between NLRP3 and NEK7 and inhibiting the assembly and activation of NLRP3 inflammasome, exerting anti‐inflammatory effects [127]. RRx‐001, an anticancer drug, is a highly selective and effective NLRP3 inhibitor that covalently binds to cysteine 409 of NLRP3, blocking the interaction between NLRP3 and NEK7 and alleviating symptoms of EAE [128]. Selective NLRP3 inhibitor OLT1177 significantly reduces the levels of inflammatory cytokines TNF‐α, IL‐6, and IL‐1β in the spinal cord of EAE mice [129]. The selective NLRP3 inhibitor 1,2,4‐TTB can inhibit ASC oligomerization and the interaction between NLRP3 and ASC, reducing caspase‐1 activation and IL‐1β secretion in immortalized mouse bone marrow‐derived macrophages (iBMDM) and primary murine MG, significantly improving EAE progression and demyelination [130]. Dimethyl fumarate, as a novel NLRP3 inflammasome inhibitor, can inhibit the priming and activation of NLRP3 inflammasome in MG and MФ, exhibiting therapeutic effects in MS [100, 131, 132]. IC100, the latest developed inflammasome inhibitor, is a humanized antibody targeting the ASC component of inflammasomes. It has the capability to inhibit various inflammasomes, including NLRP1, NLRP3, NLCR4, and AIM2. In a mouse EAE model, IC100 inhibits ASC, reduces CD4 and CD8 T cell infiltration in the spinal cord, and improves EAE progression and severity [133]. This novel antibody therapy can also decrease microglial activation, a hallmark of MS pathogenesis and progression [134, 135]. Furthermore, caspase‐1 cleavage is a crucial step leading to the secretion of IL‐1β and IL‐18, and targeting caspase‐1 can also block the activation of NLRP3 inflammasome. The small molecule inhibitor VX‐765 can covalently modify the Cys285 of caspase‐1, effectively inhibiting caspase‐1 activity and abnormal NLRP3 inflammasome activation in MS model MФ and MG [101]. Saito et al. demonstrated that intranasal delivery of VX‐765 therapy is a promising treatment approach for progressive MS and other neuroinflammatory diseases [136]. GSDMD represents the final and bottleneck step in all inflammasome activation and functions, making it an attractive target for drug development. It has been reported that the small molecule inhibitor disulfiram can upregulate miR‐30a expression to inhibit GSDMD levels, suppress Th17 differentiation, and improve EAE [137]. A recent study further revealed that disulfiram covalently modifies Cys191/Cys192 in GSDMD, preventing pore formation and inhibiting IL‐1β release and pyroptosis [138]. In summary, there is extensive research on the treatment of NLRP3 inflammasome‐related pathways. However, there are few drugs currently available for clinical use, and there is no effective cure for MS. Therefore, further research is necessary to investigate NLRP3 inflammasome‐related pathways and provide clues for the treatment of MS patients.

### 7.2 Targeting Innate Immune Cells MG and MФ for MS Treatment

A wealth of literature has reported a range of beneficial and detrimental effects of MФ and MG in MS models, yet there is still a lack of specific drugs targeting these innate immune cells. Recently successful approaches for treating SPMS include the oral treatment drug, Siponimod, an S1P receptor modulator; the B‐cell targeted therapy drug ocrelizumab; and the selective immune reconstitution therapy drug cladribine [77]. Siponimod, an orally administered FDA‐approved drug for treating active SPMS, selectively binds to S1P1 and S1P5 receptors, which are expressed in cells of the central nervous system, including MG, astrocytes, and MG [139, 140]. It can attenuate the release of the MG cytokines IL‐6 and RANTES [140]. Other S1P receptor modulators used for MS treatment include fingolimod and ozanimod, but fingolimod is only applicable to RRMS as its efficacy in SPMS was not confirmed in relevant Phase III clinical trials [141]. Ozanimod has similar target receptors (S1P1 and S1P5) as Siponimod and can reduce the expression of pro‐inflammatory cytokines in MФ and MG, which may explain its potential neuroprotective effects [142]. Ocrelizumab, a B‐cell‐depleting agent, is used for treating RRMS and PPMS. In the Phase III clinical trial (ORATORIO), the safety and efficacy of ocrelizumab in PPMS were confirmed; the treatment group using ocrelizumab had a 24% lower risk of confirming disability progression at 12 weeks compared to the placebo group [143]. Cladribine is a selective immunosuppressant used for treating RRMS and active SPMS. In addition to lymphocyte clearance, cladribine can also directly act on CNS cells by crossing the blood–brain barrier; for example, studies on primary MG cultures treated with cladribine have shown a reduction in MG granularity, phagocytic ability, and changes in gene expression, indicating lower activation levels of MG [144, 145]. Moreover, cladribine can induce apoptosis in MG cultures [144].

Currently, ongoing clinical trials are evaluating several potential treatment options for SPMS, such as immune‐modulating therapies targeting bone marrow cells (e.g., dimethyl fumarate, masitinib, sulforaphane) or potential neuroprotective agents (e.g., simvastatin) [77]. Research has shown that dimethyl fumarate can reduce the number of infiltrating and activated MФ/MG in the CNS, as well as proliferating astrocytes (GFAP), thus improving the severity of EAE [146]. After 1 week of treatment, dimethyl fumarate can reduce MG activation and promote downregulation of Iba‐1 in the focal‐delayed type hypersensitivity rat model of MS (fDTH‐EAE) [147, 148]. However, a randomized controlled trial investigating the effect of dimethyl fumarate on PPMS patients for 48 weeks found no impact on any study efficacy measures [149]. Considering that MФ/MG mainly generates oxidative stress during EAE, antioxidants may be a way to limit potential toxicity mediated by ROS [149]. Sulforaphane can induce changes in MG actin, thereby altering their phagocytic ability, motility, and migration, leading to a slowdown in the progression of MS [150]. Currently, an ongoing Phase II trial will involve over 100 patients to evaluate the impact of sulforaphane on the functional capacity and brain volume of SPMS patients, with results expected to be published in 2024 (ClinicalTrials.gov NCT03161028).

Masitinib is a selective tyrosine kinase inhibitor that can alleviate neuroinflammation by regulating the survival, migration, and degranulation of mast cells [151], which may contribute to the development of MS [152]. Masitinib also inhibits the key receptor CSF1R that promotes MG proliferation [153, 154]. Simvastatin, an HMG‐CoA reductase inhibitor or statin drug, can protect the central nervous system of SPMS by inhibiting MG activation, suppressing pro‐inflammatory mediators (such as TNF‐α, IL‐1β, IL‐6, ROS, IFN‐γ, COX‐2, PGE2, and RNS), and promoting the release of the immune‐regulatory cytokine IL‐10 [155]. Currently, a Phase III clinical trial is underway, involving over 1000 SPMS patients, to investigate the impact of simvastatin on disability progression, with an estimated completion date of August 2024 (ClinicalTrials.gov NCT03387670).

Currently, drugs used for the treatment of MS also include glucocorticoids and interferons. For example, in a demyelinating mouse model, prednisone can inhibit the activation of the inflammasome signaling pathway, as well as levels of related inflammatory cytokines and chemokines, and reduce MG activation [156]. A Phase II clinical trial involving 30 MS patients is evaluating the effect of prednisone on acute inflammatory lesions at the edges of MS plaques, with an estimated completion date of December 2028 (ClinicalTrials.gov NCT02784210). However, the outcomes of using β‐interferon therapy differ for SPMS and PPMS. β‐interferons can reduce the relapse rate in SPMS patients, but clinical trials have not demonstrated their beneficial effects on disability progression in PPMS [77]. Overall, the use of treatment strategies targeting MG and MФ with multiple mechanisms brings new hope for patients with MS.

## 8 Conclusion and Outlook

In conclusion, the activation of NLR, particularly NLRP3 inflammasome, in MG and MФ plays a significant role in the progression of MS. On the one hand, the activation of the inflammasome promotes neuroinflammation, leading to worsening of the pathological changes in MS. On the other hand, the activation of the inflammasome may also accelerate the progression of MS and EAE neuroinflammation and demyelination by promoting reactivity and phenotypic transformation in MG and MФ.

In recent years, there have been many new developments in the treatment of MS. For instance, AI‐based drug discovery technology has gradually been applied in the research of MS therapy. This technology can quickly identify potential drug molecules or biomarkers through big data analysis. Additionally, the modeling effectiveness of the MS model EAE is poor for SPMS. Therefore, expanding the MS model may help address the challenges in SPMS research. The recently developed zEAE model, which is based on zinc uptake deficiency in mice, provides a better simulation of the progression of SPMS.

Furthermore, various NLRP3 inhibitors have been developed and entered early clinical trials. In addition, other family members, such as NLRP1 and NLRP6, have shown important roles in certain inflammatory diseases, and drugs specifically inhibiting these proteins are being developed. Based on the above understanding, targeting NLRP3 inflammasome and innate immune cells MG and MФ may become an effective means to improve the progression of MS. However, the effectiveness and safety of this strategy still need to be further validated in large‐scale clinical trials.

At the same time, we also need a deeper understanding of the activation mechanism of NLR inflammasome and how it affects the functions of MG and MФ. This will provide new insight for the development of more effective treatment strategies.

## Author Contributions

Hua Fan, Qizhi Fu, and Ganqin Du contributed equally to the conception and design of the study. Hua Fan, Dongmei Wang, and Yanhui Yang supervised the project and were responsible for data interpretation. Qizhi Fu, Ganqin Du, and Ling Qin performed the literature search and drafted the manuscript. Xiaofei Shi and Dongmei Wang reviewed and critically revised the manuscript for important intellectual content. All authors read and approved the final version of the manuscript.

## Ethics Statement

The authors have nothing to report.

## Conflicts of Interest

The authors declare no conflicts of interest.

## Data Availability

All data can be provided as needed.
